# Debunking the Myth of Exercise-Induced Immune Suppression: Redefining the Impact of Exercise on Immunological Health Across the Lifespan

**DOI:** 10.3389/fimmu.2018.00648

**Published:** 2018-04-16

**Authors:** John P. Campbell, James E. Turner

**Affiliations:** Department for Health, University of Bath, Bath, United Kingdom

**Keywords:** exercise, physical activity, upper respiratory tract infections, open window hypothesis, infection susceptibility, ageing, immunosenescence, immune competency

## Abstract

Epidemiological evidence indicates that regular physical activity and/or frequent structured exercise reduces the incidence of many chronic diseases in older age, including communicable diseases such as viral and bacterial infections, as well as non-communicable diseases such as cancer and chronic inflammatory disorders. Despite the apparent health benefits achieved by leading an active lifestyle, which imply that regular physical activity and frequent exercise enhance immune competency and regulation, the effect of a single bout of exercise on immune function remains a controversial topic. Indeed, to this day, it is perceived by many that a vigorous bout of exercise can temporarily suppress immune function. In the first part of this review, we deconstruct the key pillars which lay the foundation to this theory—referred to as the “open window” hypothesis—and highlight that: (i) limited reliable evidence exists to support the claim that vigorous exercise heightens risk of opportunistic infections; (ii) purported changes to mucosal immunity, namely salivary IgA levels, after exercise do not signpost a period of immune suppression; and (iii) the dramatic reductions to lymphocyte numbers and function 1–2 h after exercise reflects a transient and time-dependent redistribution of immune cells to peripheral tissues, resulting in a heightened state of immune surveillance and immune regulation, as opposed to immune suppression. In the second part of this review, we provide evidence that frequent exercise enhances—rather than suppresses—immune competency, and highlight key findings from human vaccination studies which show heightened responses to bacterial and viral antigens following bouts of exercise. Finally, in the third part of this review, we highlight that regular physical activity and frequent exercise might limit or delay aging of the immune system, providing further evidence that exercise is beneficial for immunological health. In summary, the over-arching aim of this review is to rebalance opinion over the perceived relationships between exercise and immune function. We emphasize that it is a misconception to label any form of acute exercise as immunosuppressive, and, instead, exercise most likely improves immune competency across the lifespan.

## Introduction

Lifelong physical activity^1^ is a potent means of reducing the risk of non-communicable diseases including cancer, cardiovascular disease, and other chronic inflammatory disorders (1). Evidence also shows that a physically active lifestyle diminishes the risk of contracting a range of communicable diseases including viral and bacterial infections (2–6). In contrast to the widely accepted long-term health benefits that are achieved by regular physical activity, which imply that immune competency and regulation are improved by frequent exercise bouts, the effect of a single bout of exercise on immune function remains hotly disputed. Undeniably, acute vigorous exercise has a profound effect on the phenotypic makeup and functional capacity of the immune system. Indeed, the behavior of almost all immune cell populations in the bloodstream is altered in some way during and after exercise (7, 8). However, for decades, it has been widely accepted that these changes result in a temporary decline in immune competency in the hours following exercise. In the first part of this review, we re-interpret these data and strive to dispel the misconception that an acute bout of exercise is detrimental to immunological health. In the second part of this article, we demonstrate that rather than suppressing immunity, contemporary evidence shows that an acute bout of exercise improves immune surveillance, for example leading to enhanced antibacterial and antiviral immunity. Finally, in the third part of this article, we summarize recent data suggesting that regular physical activity and frequent exercise, which reduces systemic inflammatory activity and improves aspects of immune function, also leads to alterations in classical biomarkers of an aging immune system. These changes could be interpreted as limiting or delaying immunological aging (9–13).

## Part A: Is it Time to Close the Shutters on the “Open-Window” Hypothesis? A bout of Exercise Does Not Suppress Immune Competency

A prevailing myth has formed in the literature that participating in an acute bout of aerobic exercise, particularly if it is vigorous and prolonged, can be detrimental to immune competency. The foundations of this belief lay in research publications emanating from the 1980s and 1990s, reviewed extensively elsewhere (7, 8, 14). Findings from these early studies led to three principles of exercise immunology being formed, which have, until now, generally been unchallenged in the literature: (i) infection risk is increased after an acute bout of prolonged and vigorous aerobic exercise; (ii) acute bouts of vigorous exercise can lead to a temporary reduction to salivary IgA levels culminating in a higher risk of opportunistic infections; and (iii) transient decreases in the number of peripheral blood immune cells, which occurs in the hours following vigorous exercise, represents a period of immune suppression. Over the years, these collective observations have coalesced and led to the creation of the so-called “open-window” hypothesis, which purports that the immune system is compromised in the hours after vigorous exercise, leading to an increased risk of opportunistic infections in the days thereafter. To this day, the “open window” hypothesis continues to be discussed (15), despite the existence of contradictory evidence. Here, in the first part of this review, we aim to dispel the “open-window” hypothesis by revisiting key research studies and highlighting that limited evidence exists to support each of the three pillars that lay its foundation.

### Exercise and Opportunistic Infections

It has been speculated for over a century that participation in physical activity heightens the risk of opportunistic infections (16). However, deeper investigation into the relationship between exercise and infection susceptibility did not take place until the end of the twentieth century. At this juncture, the principle focus of many of these studies in the late 1980s and early 1990s was to determine whether infection incidence was increased in elite and recreational athletes in the weeks following mass participation distance running events. One of the first studies from this era found that one third of 150 runners participating in the 1982 Two Oceans 56 km ultramarathon in Cape Town South Africa self-reported symptoms of upper respiratory tract infections (URTIs; symptoms = runny nose, sore throat, sneezing) within 2 weeks of the race (17). The control group, who were age matched and shared a home with another of the race competitors, reported only half the amount of URTIs in the same period (17). Similar observations were made in an often-cited larger study of the 1987 Los Angeles Marathon (18). Of 2,311 respondents who had completed the marathon and whom did not report an infection in the week prior to the race, 12.9% reported an infection in the week after the race compared to only 2.2% of individuals who withdrew from the race for reasons other than illness (odds ratio of infection in runners versus non-runners = 5.9). A separate study conducted around the same time found that shorter duration running events, such as 5, 10 km, and half-marathons (21 km) did not appear to elicit an increased incidence of self-reported URTIs (19), thereby suggesting that URTI symptoms are increased only when the duration of the exercise is long.

A fundamental limitation of each of the aforementioned studies is that none of the self-reported infections were clinically confirmed by laboratory analyses (e.g., molecular or microbiological techniques, such as polymerase chain reaction or bacterial cultures). As a consequence, it was questioned whether the self-reported URTIs in these studies represented genuine infections. Clarifying this issue, a study employing nasopharyngeal and throat swabs in athletes who reported URTI symptoms over a 5-month period—including periods of competition—found that few of the self-reported infections were of bacterial, viral, chlamydial, or mycoplasmal nature (20). Indeed, of 37 episodes of URTI reported by athletes in this study, only 11 of these (30%) had a positive laboratory diagnosis. These findings place the previously discussed marathon studies in a different light (17, 18) and open the possibility that many of the URTIs reported were a symptom of other non-infectious causes. Indeed, of the of the non-infectious “URTIs” reported by Spence et al. (20), and likely captured elsewhere (17, 18), it is proposed that these symptoms are a result of other causes, including allergy and asthma, non-specific mucosal inflammation, or airway epithelial cell trauma due to increased ventilation or exposure to cold air (21). In the few cases of clinically confirmed URTIs, these appear to be from viruses—in particular rhinoviruses (i.e., the “common cold”)—rather than bacterial infection (20, 22), which is in line with the typical incidence and etiology of infections at the population level (23).

In the previously discussed clinical laboratory study of infection incidence in athletes (20), it is notable that the proportion of athletes with a confirmed infection during the 5-month period was as follows: 2/20 controls (10%), 3/31 (10%) recreational athletes, and 6/32 (19%) elite athletes. Although this study was small, these data appear to align with observations from earlier self-report studies which found that the incidence of URTI symptoms was higher in those with the fastest race time and those who had completed a greater training volume pre-race (17, 18). These early observations contributed to the formulation of the “J-shaped curve” (24). This hypothesis infers that those who undertake an excessive volume of exercise, over a period of weeks and months, sometimes referred to as “over-training” or “intensified training” (25, 26), are at a greater risk of infections (24). In this scenario, other factors present *prior* to an acute bout of exercise, such as psychological stress and anxiety (27–29), or nutritional deficiencies (30) which are known to impact immune regulation, are likely to impact immune competency and contribute to the risk of genuine URTIs, rather than the acute and transient immune changes that arise *after* the acute bout of exercise itself; these acute immunological changes arising after acute exercise are discussed later in this article (see Part A: “Is it Time to Close the Shutters on the “Open-Window” Hypothesis? A Bout of Exercise Does Not Suppress Immune Competency”; and see “Exercise and Salivary IgA and Changes to Lymphocyte Frequency and Functional Capacity in the Hours After Acute Exercise”).

Moreover, we contend that attendance at any mass participation event—whether it is a marathon or otherwise—is likely to increase the risk of acquiring novel infectious pathogens, which are in abundance due to the mass gathering of people. For example, it has been shown that around 40% of individuals attending the Hajj—a crowded religious event in Saudi Arabia—self-report an URTI (31). In this study, there was a greater risk of infection among those with the longest exposure to crowds (31). Thus, it is important to consider that other underlying factors, often not measured in the context of exercise and illness studies, likely play a greater role in infection risk than exercise participation *per se*. For example, recently, it has been demonstrated that air travel is a significant predictor of illness symptoms in athletes (32). Infections linked to air travel are exacerbated by long-haul flights crossing multiple time zones, implicating many other factors known to influence immune function, including exposure to hypobaric hypoxia, radiation, temperature changes, sleep disruption, fatigue, altered or inadequate diet, dehydration, and psychological stress (32–34).

Contrary to the aforementioned reports that exercise heightens infection incidence, it is often overlooked that other studies indicate that exercise participation may in fact *reduce* the incidence of infections. For example, a recent prospective cohort study of 1,509 Swedish men and women aged 20–60 years found that higher physical activity levels were associated with a lower incidence of self-reported URTIs (35). A much smaller but very detailed analysis of illness records kept by 11 elite endurance athletes over a period of 3–16 years showed that the total number of training hours per year was inversely correlated with sickness days reported (36). Similarly, another study of swimmers monitored for 4 years found that national level athletes had higher incidence of infections than more elite international level athletes (37). Finally, studies of ultramarathon runners, who undertake the largest volume of exercise among athletes, have shown that these individuals report fewer days missed from school or work due to illness compared to the general population. For example, the mean number of sickness days reported over 12 months was 1.5 days in a study of 1,212 ultramarathon runners and 2.8 days in a study of 489 ultramarathon runners (38, 39). These studies compared their findings to data from the United States Department of Health and Human Services report in 2009, showing that the general population report on average 4.4 illness days each year. Thus, a number of studies challenge the “J-shaped curve,” indicating that athletes undertaking the largest training loads, become ill less frequently than athletes competing at, and training at, a lower level. These findings have previously been conceptualized by extending the “J-shaped curve” into an “S-shaped curve,” thereby suggesting that very elite athletes are better adapted to the demands of their training (40). Given the nature of their design, very few of these reports—akin to many of the aforementioned studies showing increased infection risk among athletes following mass participation endurance events—used appropriate laboratory diagnostics to confirm an infection. However, despite their limitations, it is important to highlight that there are as many epidemiological studies showing that regular exercise *reduces* infections as there are studies showing exercise *increases* infections, and that these studies are often overlooked in the exercise immunology literature.

It should also be considered whether the commonly reported “increased frequency” of illness symptoms among athletic populations or those taking part in sporting events is indeed more frequent than among the general population. For example, large studies have reported that approximately 7–10% of athletes competing in the Olympic Games report symptoms of illness during the competition weeks (41, 42). However, accumulating evidence suggests the incidence of infection among athletes is not substantially different to other populations. For example, in a telephone survey of 2011 adults considered to represent the general population in the USA, 24% experienced a cold during a 4-week period, which is a similar timeframe to many international sporting competitions (43). In another telephone survey of 4,051 adults in the USA, 72% experienced at least 1 non-influenza related URTI over 12 months, and on average, experienced 2 infections annually (44). In a year-long Internet-based monitoring study of 627 individuals over 14 years of age in Germany, weekly acute respiratory illness rates were 2.7–8.2%, manifesting in 1.3–3.2 episodes annually (45). Thus, evidence suggests that the frequency of illness episodes in athletic communities is similar to the general population annually.

In the context of exercise participation in older age, it would appear to be counterintuitive that the incidence of URTI symptoms appears to be inversely correlated with age: it has been shown that URTI symptoms are more common in younger rather than older runners (18). While, once more, this aforementioned study did not confirm infections by laboratory analyses, it is notable that if acute exercise does suppress immune competency, it might be expected that older adults—whom typically have inferior immune function—would be at greatest risk of exercise-induced immune suppression. Rather than exercise *per se*, again, a more likely explanation for differences in illness symptoms between groups of athletes are other factors present before competing, such as fatigue, nutritional deficiency, psychological stress, or environmental exposures. On the other hand, proponents of the “open-window” hypothesis could portend that experienced athletes have higher tolerance of the symptoms associated with URTI, and/or have developed coping methods or strategies to reduce symptoms. Alternatively, experienced athletes may have evolved strategies to reduce infection risk by adopting good practice (e.g., sleep, diet, hygiene) before, during and following attendance at a mass participation event.

Separately, it has also been questioned whether illness symptoms—even if confirmed to be infectious—are a result of encountering a novel pathogen. For example, over a decade ago, some studies suggested that reactivation of latent viruses—such as *Epstein Barr Virus* which had most likely infected the host during childhood—was responsible for illness symptoms after exercise (46, 47). Although these studies suggest new pathogens were not to blame, it was interpreted that exercise-induced immune suppression had resulted in loss of viral control. However, herpes viruses can reactivate even with a fully functioning immune system, for example, in response to adrenergic activity, reactive oxygen species and inflammatory cytokines (48–50), all of which increase during exercise. Moreover, recent evidence appears to discount herpes virus involvement in causing symptoms of illness, by showing that individuals previously infected with *Cytomegalovirus*, or those infected with both *Epstein Barr Virus* and *Cytomegalovirus*, exhibited a lower incidence of illness symptoms than individuals not latently infected (51).

Taken together, evidence that participation in an acute bout of vigorous exercise leads to heightened infection incidence remains spurious. If symptoms of URTI are observed after a bout of vigorous exercise, the cause is unlikely to be infectious. However, if infection or immune impairment is confirmed, their trigger is more likely to be the physical, nutritional, and psychological wellbeing of the individual prior to undertaking the single bout of acute vigorous exercise. In the context of mass participation sporting events, it is likely that increased exposure to pathogens, or the influence of environmental factors that can affect immune function (e.g., travel, sleep disruption) most likely explain genuine infections. Thus, we conclude that it is unlikely that vigorous and prolonged exercise heighten the risk of infections and should not be considered a deterrent to those seeking to become more physically active.

### Exercise and Salivary IgA

A second mainstay of exercise immunology that has received considerable attention over the last three decades is the assessment of exercise-induced changes to mucosal immunity, principally *via* measurement of IgA levels in saliva (52). Given that IgA is the most abundant immunoglobulin in mucosal secretions and that its principle role is the inhibition of invading pathogens, isolated changes to salivary IgA following exercise has been considered of some importance in light of the purportedly higher risk of infections among athletes (7, 8).

One of the earliest and most cited papers in this research area found that IgA was reduced by 20% after 2–3 h of cross-country skiing (53). Another study found that this effect is transient, whereby salivary IgA concentrations decreased immediately after 2 h of intensive cycling exercise, and remained low in samples collected 1 h post-exercise, but returned to normal levels within 24 h (54). A criticism of these studies at that time was that the absolute IgA levels reported did not adequately control for the amount of saliva produced, and thus these results may misrepresent IgA secretion. Although some studies measuring IgA secretory rate (IgA protein concentration multiplied by saliva flow rate) support the early findings with IgA concentration, others have shown profoundly contradictory results. For example, in alignment with prior observations, it was found in trained runners that IgA secretion rate decreased by 25% from pre-marathon to 90 min post-marathon (55). Likewise, in a separate study, a 20% reduction in IgA secretion rate was observed in elite athletes after a 2-h rowing exercise session (56). Several other studies of similar design reported analogous findings (57–59); however, contradictory findings are also in abundance but are much less cited in the literature. Indeed, an elegant study exploring the effects of different exercise intensities, including moderate- and high-intensity exercise to exhaustion, found that although saliva flow rate decreased, IgA secretion rate actually increased in response to both of the exercise bouts. In the words of the authors, exercise to exhaustion has an “*effect on the quantity of saliva, but not the quality of saliva*” (60). Many other studies have also reported that exercise does not elicit a decrease in IgA secretion rates following exercise (61–66).

Any subtle isolated changes to IgA that occur after exercise appear to be clinically insignificant as it would appear that the increased incidence of URTI symptoms that has been purported following vigorous and prolonged exercise is unrelated to salivary immunoglobulin status. A longitudinal field study of participants in the Comrades Marathon (86.5 km ultramarathon) in South Africa found that salivary IgA levels in the 4 weeks prior to the race, and 2 weeks following the race, were unrelated to the incidence of self-reported URTI (67). In the aforementioned study, it was found that symptoms of URTI were highest 4 weeks prior to the marathon, and URTI symptoms seemed to re-appear within many of the same athletes 1 or 2 weeks post-marathon. This study did not confirm the re-emergence of infections by laboratory testing, but if this was indeed demonstrated in future studies, it again shows that acute exercise participation *per se* does not heighten risk of opportunistic infections. In this case, an underlying infection, not resolved prior to exercise participation, or some other idiosyncrasy, is perhaps to blame. Such conclusions appear to be supported by evidence from athletes who report the most frequent illness symptoms. For example, these individuals exhibit mostly anti-inflammatory cytokine responses when whole blood, collected at rest, is cultured *ex vivo* with antigens from diphtheria, tetanus, acellular pertussis, poliomyelitis, and hemophilus influenza type b (68, 69). These findings suggest the immune system may be functionally altered by underlying illness, or is already different in these “illness-prone” athletes prior to infection, rather than exercise affecting immune function *per se*.

A flaw in studies investigating the link between mucosal immunity and purported exercise-induced infection risk is that oral health status is rarely adequately evaluated. Salivary IgA is heavily involved in host-bacterial ecology and mucosal homeostasis (70, 71). As optimal oral health is rare in adults—with nearly all exhibiting caries, gingivitis or periodontitis—profound between-person IgA variation has been reported, which is dependent on oral health status (71). Moreover, periodontal diseases are complex and multifactorial, and as a result, studies report large fluctuations in IgA levels relative to disease status between persons, probably due to the bespoke ecological makeup of different host mucosa (71). In addition, oral disease is a common problem in athlete populations (72), which is likely to be caused by high volume and frequent carbohydrate consumption, and in some, a neglect of oral hygiene, perhaps due to practical constraints. Thus, changes to oral inflammatory status has not been adequately considered, and emerging salivary biomarkers of oral inflammation, such as immunoglobulin-free light chains (73), may offer a means of controlling for this confounder. As highlighted elsewhere (70), salivary IgA is also highly vulnerable to short-term variation, in particular, due to circadian rhythms, typically peaking in the morning, and falling thereafter (74). As salivary IgA secretion is controlled by the parasympathetic and sympathetic nervous system, psychological stress also plays a powerful role in regulating IgA levels (75). Animal models suggest that salivary IgA levels could vary up to 27-fold within the same host over a short period of time (76). Salivary IgA levels are also affected by factors such as sex differences (77), diet, ethnicity, disease, medications, tobacco, and phase of the menstrual cycle, as reviewed elsewhere (71). To overcome some of these variations in salivary IgA, it is often the case that studies evaluate only secretory IgA (sIgA; i.e., IgA containing the secretory component) as this represents IgA produced by local mucosal plasma cells, and not IgA from the bloodstream transported *via* crevicular fluid. While this approach may reduce some confounding error, most IgA in saliva contains the secretory component and is, itself, subject to large variation; extensively reviewed elsewhere (78). Given these many considerations, we propose that longitudinal measurement of salivary IgA, as an isolated measure of immune competency within a single host, and even more so between persons, depicts too confusing a picture, and it is ambitious to say that any subtle changes to salivary IgA following exercise reflects immune suppression and a heightened risk of opportunistic infections. Given the limitations of salivary IgA measurement, research is being undertaken to explore mucosal IgA in other biofluids, and a recent study has shown links between reduced tear IgA levels and infection incidence (79). Others have moved toward more comprehensive oral immunity panels (80), and such strategies could benefit further from an integrative approach that, in addition to immune parameters, incorporates full dental examination, oral inflammation biomarkers, and host mucosal ecology.

### Changes to Lymphocyte Frequency and Functional Capacity in the Hours After Acute Exercise

One of the most reproduced findings in human exercise physiology is the profound and transient time-dependent change that arises to the phenotypic composition and functional capacity of lymphocytes in the peripheral bloodstream in response to a single bout of exercise (8). During vigorous aerobic exercise, it is commonly observed that peripheral blood lymphocyte frequency—and, concomitantly, the functional capacity of the lymphocyte pool—is dramatically increased, leading to the concept that, during exercise, exercise appears to “stimulate” the immune system. On the other hand, in the hours following exercise, it is typically observed that total peripheral blood lymphocyte frequency—and the functional capacity of the lymphocyte pool—is decreased below pre-exercise levels, leading some to propose that exercise induces a short-term window of immune suppression (termed the “open-window” hypothesis). The purpose of this part of our review is to outline that it is a misconception to state that the “reductions” to lymphocyte frequency and function, that arise in the hours following acute exercise, reflects immune suppression, and instead we emphasize that during this post-exercise period, the immune system is in a heightened state of immune surveillance and regulation.

#### Transient Changes to Blood Lymphocyte Frequency in the Hours Following Exercise

The classic biphasic response of lymphocytes to acute steady state vigorous exercise lasting for around at least 45–60 minutes, is first characterized by a dramatic lymphocytosis. This response is typified by a dramatic influx of natural killer cells, which rise by up to 10-fold, and CD8^+^ T cells which increase to a lesser—but still profound—extent by approximately 2.5-fold (81). This exercise intensity-dependent mobilization is driven in part by increased shear forces and blood pressure during exercise causing a non-specific flushing of the marginal pools (82) but, moreover, is principally governed by adrenergic stimulation of beta-2-adrenergic receptors on the surface of lymphocytes, arising from adrenaline released during exercise, causing endothelial detachment and subsequent recirculation of lymphocytes into the bloodstream (83–85). Indeed, the lymphocyte mobilization response observed during exercise appears to broadly mirror the differential expression of beta-2 adrenergic receptors on lymphocytes: natural killer cells > CD8^+^ T cells > B cells > CD4^+^ T cells, including regulatory T cells (81, 86–88). Upon exercise cessation, the classic biphasic exercise response is next characterized by a dramatic decrease in the frequency of lymphocytes in the bloodstream. This nadir is typically observed approximately 1–2 h post-exercise when the lymphocyte numerical count is lower than pre-exercise levels; lymphocyte frequency normally returns to pre-exercise levels within 24 h (87, 89, 90). The lymphopenia that occurs 1–2 h later is exercise intensity dependent and the most profound reductions during this period are typically observed among natural killer cells and CD8^+^ T cells (90). Akin to purported reductions to salivary IgA, discussed earlier, it was perceived that the numerical decline of blood lymphocytes that arises during this time represented “double jeopardy” (89), temporarily exposing an individual to impaired immune competency and concomitantly providing an “open-window” for opportunistic infections (91, 92).

Rather than suppressing immune competency however, a more contemporary viewpoint is that this acute and transient lymphopenia 1–2 h after exercise is beneficial to immune surveillance and regulation. Indeed, in what appears to be a highly specialized and systematic response, it is widely proposed that exercise redeploys immune cells to peripheral tissues (e.g., mucosal surfaces) to conduct immune surveillance. Here, these immune cells are thought to identify and eradicate other cells infected with pathogens, or those that have become damaged or malignant, termed the acute stress/exercise immune-enhancement hypothesis (93). A seminal study by Kruger and colleagues, using fluorescent cell tracking in rodents, found that T cells are redeployed in large numbers to peripheral tissues including the gut and lungs, and to the bone marrow following exercise (84, 94). In line with Dhabhar’s theory, it is hypothesized that this redistribution reflects heightened immune surveillance in sites where pathogens are likely to be encountered during and after exercise (i.e., lungs, gut). This response has also been proposed to maintain immune homeostasis *via* augmented regulatory activities (12). In this context, evidence implies that bone marrow homing and subsequent apoptosis of senescent T cells stimulates the production or mobilization of new progenitor cells into the periphery (95), which has been hypothesized as an exercise-induced means of maintaining a younger immune system (12), discussed later in Part C “Does Exercise and Regular Physical Activity Influence Immunological Ageing.” Links between exercise-induced apoptosis and lymphopenia have in the past been interpreted as detrimental, with speculation that apoptosis could be responsible for the fall in lymphocyte number in the hours after exercise (96, 97). Other studies have reported increased lymphocyte apoptosis immediately after exercise (i.e., as a result of the large mobilization of cells) but not in the hours following exercise during lymphopenia (98–100). Although the magnitude of lymphocyte apoptosis reported in studies is dependent on the measurement technique, typically <10% of lymphocytes undergo post-exercise apoptosis (96, 97). Given the 30–60% decrease in lymphocyte numbers post-exercise (89, 101, 102), apoptosis could be a small contributor to exercise-induced lymphopenia, but this process of cell death is likely to be beneficial given the stimulation of progenitor cells from the bone marrow (95).

While it has not been shown in humans that exercise—in line with rodent models—causes the redistribution of immune cells to peripheral tissues, further support for a coordinated, exercise-induced immune surveillance response elicited by lymphopenia, is revealed by studying the phenotypic characteristics of the cells that preferentially mobilize and subsequently extravasate out of the circulation after exercise. With regard to natural killer cells—the most exercise-responsive lymphocyte subset—CD56^dim^ cells are preferentially redeployed rather than their CD56^bright^ counterparts (81). CD56^dim^ cells are a mature subset of natural killer cells which have exclusive migratory potential for non-lymphoid tissue and potent effector capabilities, including the capacity to produce high amounts of perforin and granzyme, whereas CD56^bright^ cells are a more immature regulatory cell subset (103) and reside in secondary lymphoid organs, typified by their cell-surface expression of CD62L and CCR7 (104). CD56^dim^ natural killer cells can be further dissected, into cells with highly potent effector function based on loss of NKG2A and expression of killer immunoglobulin-like receptors and CD57 (105, 106). In a recent study, it was shown that these natural killer cells, which are capable of rapid effector functions, are preferentially redistributed after exercise (107, 108). Synergistically, T cells also appear to exert heterogeneous but highly coordinated responses to acute exercise. Indeed, it is consistently observed that discrete populations of CD8^+^ but not CD4^+^ T cell subsets are redeployed by exercise. For some time, there was confusion pertaining to the exact behavior of CD8^+^ T cells in response to exercise. Rather than losing or gaining markers of adhesion or activation—as evaluated elsewhere (8)—these changes represent a uniform redeployment of a preferentially mobilized group of memory cells. In a flurry of studies about a decade ago, it was shown that exercise selectively mobilizes memory CD8^+^ T cells with a phenotypic propensity for homing to peripheral tissues—typified for example by CD11, and not CCR7 or CD62L expression—and the distinctive capacity to mount rapid effector functions (81, 109–111). This response presumably facilitates the detection and elimination of neoplastic, stressed or infected cells in synergy with natural killer cells, as proposed elsewhere (112). Aligned with the immune surveillance theory of Burnet and Thomas, reviewed elsewhere (113), these results imply that sentinel cells of the immune system are redeployed by exercise-induced perturbations to stress hormones, to exert effector functions against neoplastic, stressed, or infected cells in the hours following exercise. This process, which occurs daily in a natural diurnal process (114), orchestrated subtly by stress hormones (115, 116), appears to be primed in response to exercise, leading to enhanced immune surveillance (117). Principles of the acute stress/exercise immune-enhancement hypothesis continue to be investigated. For example, it has recently been shown that acute exercise does not preferentially mobilize CD8^+^ T cells and natural killer cells with the capacity for skin-homing (118). However, skin-homing in this context is a role that may be fulfilled by exercise-responsive mucosal-associated invariant T cells (119); but further work is needed in this area.

A key example illustrating how exercise-induced immune cell redistribution is beneficial to host health can be found in the rapidly emerging field of exercise oncology. Indeed, a recent seminal study demonstrated inhibition of tumor onset and disease progression across a range of tumor models in voluntarily active rodents (112). In this work, natural killer cell infiltration was significantly increased in tumors from active versus inactive rodents, leading to the conclusion that the presence of natural killer cells (but perhaps also T cells) in tumor sites, redeployed by adrenaline during exercise stress, “provides a spark” for tumor elimination, in what could be considered a form of “exercise immunotherapy” (112, 120). Importantly, administration of propranolol—a beta 2 adrenergic blocker—abolished the adrenaline-induced redistribution of immune cells, and nullified the anti-tumor effect of exercise on neoplastic growth (112). While these studies are limited to rodents, there is growing evidence that exercise may promote anti-cancer effects in humans. For example, in a key study recently conducted in humans, it was shown that natural killer cells with a highly mature effector phenotype are preferentially redistributed after exercise, and have the capacity to exert augmented cytotoxicity against myeloma and lymphoma cells *in vitro* (107, 108). In light of these results, research is now being conducted to harness the beneficial impact of acute exercise on lymphocyte kinetics for the purposes of cancer immunotherapy (121). It is beyond the scope of this review to discuss other findings in this exciting field and we briefly conclude that the aforementioned studies imply that exercise-induced lymphocytosis, and the lymphopenia that follows, is beneficial to the immune system’s capacity to identify and neutralize damaged and neoplastic cells in peripheral tissues. Furthermore, in the context of neoplastic growth, this process may be directly responsible for reduced incidence of cancer among physically active people across the lifespan (122). Further comprehensive discussion of the role of exercise and lymphocyte kinetics in anti-tumor responses can be found elsewhere (117). Clearly more research is needed in this area, and a shift in focus toward investigating the benefits—rather than purported detrimental effects—of exercise on health, is no doubt underway and will be a key focus for exercise immunologists in the coming years.

#### Transient Changes to Blood Lymphocyte Function in the Hours Following Exercise

A common misinterpretation, brought about by the exercise-induced reductions to blood lymphocyte frequency in the hours following exercise, is the observation that the functional capacity of immune cells in the peripheral blood is reduced in the hours following vigorous exercise. As measurements of cell function in peripheral blood are entirely dependent on the cells present at the time of sampling, a change to the composition of the cells in blood in the hours after exercise—as outlined in the section; “Transient Changes to Blood Lymphocyte Frequency in the Hours Following Exercise”—will consequently lead to parallel changes in overall cell function, indicated by the performance of the sampled cells being assayed. For example, among CD8^+^ T cells, subsets that exhibit strong effector function (e.g., CD45RA^+^CD27^−^CD28^−^CCR7^−^CD62L^−^CD57^+^), substantially increase during exercise (81, 123). Thus, during exercise, blood is predominantly occupied by cells capable of responding strongly (i.e., IFN-gamma production) to *in vitro* stimuli, and therefore, many studies have shown an increase in IFN-gamma production by cells isolated close to the exercise stimulus. In the hours following exercise, the same effector CD8^+^ T cells are subsequently redeployed to peripheral tissues, and, as such, this results in the blood having fewer cells capable of responding strongly to *in vitro* stimuli, thus explaining the commonly reported decrease in cellular function post-exercise. These effects have been neatly demonstrated approximately two decades ago when it was shown that IFN-gamma production by stimulated CD8^+^ T cells is reduced 2 h after completing a prolonged 2.5 h bout of cycling (124). Importantly, it was shown that this reduced capacity to produce IFN-gamma was due to a reduced number of IFN-gamma-positive CD8^+^ T cells in peripheral blood at the same time-point (124, 125).

The same principles apply to other cell functions, such as *in vitro* proliferation in response to mitogenic stimuli. However, with this measurement in particular, the commonly reported increase in T cell proliferation immediately after acute bouts of exercise is also strongly influenced by laboratory processes and *in vitro* assay conditions (e.g., blood processing, temperature, mitogen selection) (126). A recent meta-analysis of 24 studies concluded that lymphocyte proliferation is suppressed following acute bouts of exercise, and that a greater magnitude of suppression is caused by exercise lasting longer than 1 h, regardless of exercise intensity (127). However, this meta-analysis did not examine the most important determinant of cell function following exercise: the time-dependent changes in the cellular composition of the samples assayed. Thus, findings such as these should be interpreted with caution if analyses did not differentiate between studies collecting samples immediately after exercise or in the hours following.

As with research focusing on T cells, a similar group of studies citing reductions to natural killer cell cytotoxicity following acute exercise, reviewed elsewhere (128), did not always take into account dramatic shifts in the constitutional makeup of the natural killer cell compartment, which is known to change in response to exercise (81). Once more, changes to the functional capacity of the total natural killer cell pool are likely to have been misrepresented, given that many of these cells, with potent effector functions, are redistributed to peripheral tissues following exercise cessation. The principles discussed herein regarding lymphocyte function are also broadly applicable to the assessment of function in other cells, such as neutrophils and monocytes; the response of these cells to exercise is beyond the scope of this article and is reviewed elsewhere (8).

Taken together, it is important to emphasize that statements such as “*acute intensive exercise elicits a depression of several aspects of acquired immune function*” and “*prolonged bouts of heavy exertion reduce the normal functioning of all major immune cell subtypes*” mentioned elsewhere (8, 15) should be interpreted with caution. We conclude that the results of studies exploring the effects of acute exercise on cell function must be considered very carefully in light of the time-dependent changes in the cellular composition of blood that typically arise following a vigorous bout.

#### Summary: Exercise Induces Lymphocyte Immune Surveillance Not Immune Suppression

In summary, strong evidence implies that a reduction in the frequency and function of lymphocytes (and other immune cells) in peripheral blood in the hours following vigorous and prolonged exercise does not reflect immune suppression. Instead, the observed lymphopenia represents a heightened state of immune surveillance and immune regulation driven by a preferential mobilization of cells to peripheral tissues. As such, nutritional interventions, which have been employed to dampen the magnitude of exercise lymphopenia (124, 129) are unlikely to reduce the incidence of infections, but interventions that augment exercise-induced lymphocyte trafficking may provide benefits (130).

## Part B: Regular Physical Activity and Frequent Exercise Augment Aspects of Immune Competency Across the Lifespan

Contrary to a commonly held belief—outlined in Part A “Is it Time to Close the Shutters on the “Open Window” Hypothesis? A Bout of Exercise Does Not Suppress Immune Competency?”—that acute vigorous exercise can suppress aspects of immune function, an increasingly large body of research indicates that both single bouts of exercise, or frequent participation in regular exercise, can act as an adjuvant to stimulate the immune system. Numerous methods exist to assess the effects of behavioral interventions on immunity (131) but arguably the optimal means of evaluating global immune competency at a systems level is *via* assessment of the immune response to *in vivo* challenge, ideally with a novel and clinically recognized pathogen, for example *via* vaccination (132). Thus, here, in the first section of Part B “Regular Physical Activity and Frequent Exercise Augment Aspects of Immune Competency Across the Lifespan,” we focus on the influence that exercise has on immune responses to antigenic challenge, which requires a coordinated response from most, if not all, innate and adaptive immune system components. In light of the age-associated decline in immune competence with aging—caused in part by underlying changes to the numerical, phenotypic, and functional capacity of almost all innate and adaptive immune cells—the second section of Part B “Regular Physical Activity and Frequent Exercise Augment Aspects of Immune Competency Across the Lifespan,” will evaluate the impact aging has on the immune benefits that can be attained from exercise throughout the lifespan. A detailed discussion of immunological aging processes and the influence that an active lifestyle has on established biomarkers of an aging immune system is covered in Part C “Does exercise and Regular Physical Activity Influence Immunological Ageing?” of this article.

### Exercise and Immune Responses to Experimental *In Vivo* Challenge Across the Lifespan

In a research context, the most clinically relevant model to assess *in vivo* challenge in a controlled manner is *via* vaccination. Vaccine administration assesses the integrated capacity of the immune system to recognize and process antigen, leading to antigen neutralization. In a clinical research context, vaccination responses are principally quantified clinically in two ways, either *via* antibody production from antigen-specific plasma cells or *via* cytokine responses—typically IFN-gamma production—from T cells stimulated with cognate antigen.

#### Vaccination: Effects of a Single Exercise Bout

Evidence from an array of studies, evaluated recently in a comprehensive review elsewhere (9), indicates that a single acute bout of exercise appears to enhance immune responses to vaccination in both younger and older individuals. The majority of studies to date have examined muscle-damaging upper arm resistance exercise performed close to the time of vaccination which is administered shortly after the regimen into exercised muscle. However, other modes of exercise, including acute bouts of whole body aerobic activity, have also been investigated. Six of the eight trials identified in the aforementioned review indicated a statistically significant exercise-induced enhancement of immune responses against constituent antigens contained within the vaccine administered (133–138). It is notable that in five of these studies, statistically significant benefits were found where the vaccine strains appeared to have lower immunogenicity (9). For example, it was shown in a trial of 133 young adults, approximately 20 years of age, receiving either a full- or half-dose Pneumovax-23 (a pneumococcal vaccine), that those who exercised at the time of receiving the half-dose vaccine had heightened responses to five of the eleven pneumococcal strains contained in the vaccine, whereas no differences were observed for the other six strains; and no benefits of exercise were observed for the full-dose vaccine (137). As such, given the potential effectiveness of exercise as an adjuvant in situations where vaccine immunogenicity is low, studying the effects of exercise on antibody responses in older adults—whom typically exhibit impaired responses—has received considerable attention. In one recent study, it was found that antibody responses in women 55–75 years of age, were significantly improved when moderate-intensity aerobic exercise was performed immediately prior to vaccination; however, no beneficial effects were found in men (138). Another trial reported no benefits of a single 45-min bout of moderate-intensity walking exercise on the immune response to influenza and pneumococcal vaccination in women around 47 years of age (139). Finally, a very recent study found no effect of a bout of resistance exercise on antibody responses to influenza vaccination in adults approximately 73 years of age (140). It is possible that a number of factors including immunological aging, biological sex and variations in sex hormone levels, and perhaps latent infections (e.g., herpes viruses) (141–143) limit the immunostimulatory effects of exercise, and many studies have not adequately controlled for these factors. Alternatively, in light of the mechanisms proposed in the acute stress/exercise immune-enhancement hypothesis (93), it is plausible that more intensive exercise may be needed to elicit enhanced vaccine responses. Despite null findings, it is important to point out that few, if any, studies investigating the effects of acute exercise on vaccination responses have reported exercise-induced *impairment* to immune responses, and rather, these studies report that exercise has no effect, or in some cases a beneficial effect, on the immune response to vaccination in older adults.

#### Vaccination: Effects of Frequent Exercise Bouts

Data from vaccine studies exploring the effects of regular physical activity or frequent exercise training on the immune response to vaccination provides robust support for the argument that exercise enhances, rather than suppresses immunity. Indeed, at least eight studies have demonstrated heightened vaccination responses in older adults, typically over 60 years of age, who are physically active (144–151). For example, an early study categorized adults aged 62 years or older, into one of three groups: active (undertaking at least 20 min of vigorous exercise three or more times per week), moderately active (undertaking regular exercise but with lower intensity, frequency, and/or duration), or sedentary (non-exercisers). Two weeks after influenza vaccination, it was shown that serum anti-influenza IgG and IgM titers were higher in active versus sedentary adults, and so too were peripheral blood mononuclear cell responses to antigen-specific stimulation (144). In addition, a recent study has shown that men aged 65–85 years, who regularly undertook moderate or vigorous exercise training, exhibited higher antibody responses compared to inactive controls in response to an influenza vaccine (151). Data linking habitual levels of physical activity to enhanced immune competency in humans are supported by evidence from animal studies, and show that the immunological benefits of exercise may be particularly beneficial in enhancing otherwise poor responses in older age (152).

#### Interpreting Data From Vaccine Trials

A major criticism of vaccine and exercise trials conducted in humans is that many solely focus on the maximal antibody titer following vaccination, and it is not practical to follow-up with investigations into infection incidence as a gauge of protection status following vaccination (153). As such, it is unknown whether differences observed with absolute antibody titers, or the amount of IFN-gamma produced from stimulated T cells, between exercise and control groups, is representative of clinically meaningful benefits in terms of protection from infections. Three elegant studies in rodents imply that benefits, beyond antibody titer, may be brought about by acute exercise. In one of these studies, it was found that antibody responses to influenza exposure were *lower* in rodents that exercised around the time of exposure, compared to those that did not exercise, yet, exercised mice were still protected and did not exhibit any signs of infection upon re-exposure to the virus (154). Moreover, in an earlier study by the same authors, it was found that mice undertaking an acute bout of exercise before being expose to influenza exhibited a lower severity of infection and had enhanced viral clearance and lower inflammation in the lungs in the days following (155). Thus, it may be the case that exercise enhances immune responses, beyond those captured using maximal antibody titer as an endpoint. In accordance with this view, an elegant rodent study conducted by an independent group (156) found that mice exercised for 20–30 min at moderate intensity 4 h after intranasal influenza exposure had a substantially higher survival rate (18 of 22 survived; 82%) when compared to mice that did not exercise after influenza exposure (10 of 23 survived; 43%).

#### Contact Sensitivity Reactions and Acute Exercise Bouts

More recent studies have examined the effects of acute exercise on immune competency using other *in vivo* models of immune challenge that, in principle, also assess the coordinated efforts of immune system components. These studies have employed contact-sensitivity reactions by topically applying to skin, the dendritic cell and T cell stimulant (or attractant) diphenylcyclopropenone (DPCP), and the non-specific inflammatory stimulant, croton oil (157, 158). For example, in studies of young adults (approximately 20–30 years of age), by applying a primary sensitizing dose of DPCP 20 min after 2 h of moderate-intensity treadmill running, and assessing recall challenge 4 weeks later, it has been concluded that this form of exercise impairs both the induction of T cell immunity and the memory response (159, 160). Thirty minutes of moderate- or vigorous-intensity running had no effect, and no forms of exercise modulated the non-specific inflammatory challenge in response to croton oil (159, 160). Although these findings are biologically interesting, the clinical relevance of exercise-induced change is unclear, in part, because the process of DPCP-induced immune modulation is not well defined (158) unlike the immune response to antigen administration by vaccination.

#### Summary: Exercise Enhances Immune Responses to *In Vivo* Antigenic Challenge

We conclude that there is growing evidence from a powerful array of studies in humans and rodents, indicating that exercise enhances, or at least does not suppress immune responses to *in vivo* challenge in younger and older individuals. These observations—which contradict those predicated by the “open-window” hypothesis—support the contention that an acute bout of exercise has no detrimental immune consequences for health. Thus, exercise should be encouraged, particularly for older adults who are at greatest risk of infections and who may obtain the greatest exercise-induced benefits to immune competency; an overview of the impact of aging on the immunological benefits that can be attained from exercise is described next.

### Does Aging Influence the Immunological Benefits of Exercise and Regular Physical Activity?

#### Effect of a Single Exercise Bout

Research investigating the effects of exercise on immune function has sought to establish whether the observed benefits, as outlined earlier in young adults, such as exercise-induced immune cell mobilisation, that has been implicated in protection against cancer, is also applicable to older adults. For example, it has been reported that the magnitude of T cell mobilization in response to acute vigorous exercise is smaller in older (65 ± 1 years) compared to younger (22 ± 1 years) adults (161, 162). However, in this study, it was also shown that following exercise, the magnitude of T cell proliferation in response to mitogens was smaller in young adults, whereas a similar exercise-induced stimulation of natural killer cell cytotoxicity was observed for both groups (161, 162). It is beyond the scope of this review to fully critique investigations examining the influence of single exercise bouts on the function of different immune cells, with comparisons made between younger and older individuals across the lifespan; we refer the reader to comprehensive reviews on this topic (163–165). Nevertheless, it is important to point out that many studies in this area are difficult to interpret: at the time of their publication, the influence of *Cytomegalovirus* infection on the magnitude of exercise-induced immune responses was not known and was therefore not considered (123, 166). Moreover, while the magnitude of change to lymphocyte kinetics is likely to be important for detecting and eliminating viral and bacterial infections and neoplastic cells, this process is complex to study, and comparisons between younger and older people is difficult, partly due to other age-associated changes that influence the physiological response to exercise, such as the decline in fitness (e.g., sarcopenia, cardiorespiratory fitness) with age. It is likely that some, or all of these factors impact upon on the efficacy of exercise as an adjuvant to vaccination responses in older adults, outlined earlier in the section “Exercise and Immune Responses to Experimental *In Vivo* Challenge Across the Lifespan.” In light of the challenge interpreting the clinical relevance of the aforementioned lymphocyte kinetics studies, from here onward, we briefly evaluate the impact of regular physical activity or frequent exercise bouts on immune competency across the lifespan, using measurements in samples collected from participants at rest.

#### Frequent Bouts of Exercise

Immune competency at rest has been assessed in cross-sectional studies, comparing elderly individuals differentiated by physical activity level or cardiorespiratory fitness, or by examining immune function before and after structured exercise training interventions. For example, it has commonly been reported among the elderly, that the most active participants, compared to those who are least active, show the highest T cell proliferation and cytokine production in response to mitogens (163–165). Fewer studies have assessed innate immune competency, but higher natural killer cell cytotoxicity has been consistently shown among the elderly who are active compared to less active age-matched controls (163–165). Recent studies have expanded measurements into other innate immune cells such as neutrophils. For example, a recent cross-sectional study of 211 elderly adults, showed that neutrophils from the 20 most active participants, compared to the 20 least active participants, migrated toward interleukin (IL)-8 with greater chemotactic accuracy, but there were no differences in chemotactic speed (167). In addition, a recent exercise training study has shown in both young and middle-aged adults, that 10 weeks of moderate-intensity continuous cycling training, or high-intensity interval cycling training, improve neutrophil and monocyte phagocytosis and oxidative burst irrespective of age (168). Improvements in these common measurements of immune competency, however, are not always consistent in longitudinal studies employing exercise training interventions, with around half of studies reporting improvements, and half reporting no change (163–165). One reason for this could be because the dramatic effects of *Cytomegalovirus* on driving immunological aging was not considered by most of these studies, and it is feasible that results would be different when examining individuals who are latently infected compared to those who are seronegative. Importantly however, no studies report impaired immune competency from increased participation in structured exercise. Altogether, we conclude that despite declines in fitness and immune competency, aging does not appear to negate the immunological benefits that can be attained from exercise, and indeed, frequent participation in exercise across the lifespan may lead to immune benefits, even in older age.

## Part C: Does Exercise and Regular Physical Activity Influence Immunological Ageing?

Since the first exercise immunology research in the early 1900s, and the substantial increase in scientific interest from the late 1980s and early 1990s (169), studies examining interaction between immune function and lifestyle factors such as exercise and physical activity have become common. Although a few exercise studies published in the last 10–15 years have investigated some immunological processes relevant to aging, health, and disease, the theme of exercise-induced “immune suppression” continues to influence the design of new studies or at least features in the interpretation of findings and is often justified or contextualized with relevance to self-reported illness symptoms among athletes. As outlined in Part A “Is it Time to Close the Shutters on the “Open-Window” Hypothesis? A Bout of Exercise Does Not Suppress Immune Competency,” there is limited reliable evidence to show that exercise heightens risk of opportunistic infections, but there is, however, a growing body of evidence to show that exercise enhances, rather than suppresses, immune competency, as summarized in Part B: “Regular Physical Activity and Frequent Exercise Augment Aspects of Immune Competency Across the Lifespan.” The beneficial effects of exercise on immune function are likely to be greatest for elderly people exhibiting the age-associated deterioration of immune competency, also referred to as immunosenescence. Moreover, preliminary evidence suggests that physical activity and regular structured exercise may even limit or delay immunological aging. Here, in Part C: “Does Exercise and Regular Physical Activity Influence Immunological Ageing?” we evaluate whether an active lifestyle influences immunosenescence.

### The Aging Immune System

Aging is associated with profound changes to the numerical, phenotypic, and functional capacity of almost all innate and adaptive immune cells, resulting in altered immune responses. Some innate immune cells exhibit numerical, phenotypic, and functional alterations with aging, whereas others appear to be less affected (170). For example, the numbers and functions of eosinophils, basophils, and mast cells appear to be largely unchanged with aging, or at least, there is not a clear effect of age based on the limited current literature (170). Neutrophil numbers often increase with aging, but these cells exhibit diminished phagocytosis and impaired chemotaxis, although chemokinesis is maintained (170). Natural killer cells increase with aging, driven by an accumulation of cytotoxic CD56^dim^ cells but a decline in regulatory CD56^bright^ cells, and overall, cytokine production and cytotoxicity are less on a per cell basis (170). Other innate lymphocytes, such as invariant CD1d-restricted natural killer T cells (iNKT cells), which represent <1% of the T cell pool, decline in number but age-associated changes to their function have not been established (171). Monocyte numbers are stable with aging, but classical cells (CD14^++^CD16^−^) decline, and intermediate (CD14^+^CD16^+^) and non-classical (CD14^+^CD16^++^) cells increase, but overall, monocyte cytokine production is impaired (170, 172). These changes with blood monocytes are thought to be mirrored by tissue-resident macrophages, whereby classically activated M1 cells decline, and alternatively activated M2 cells accumulate (173). However, alterations in tissue-resident cells with advancing age are very likely to be a result of adipose tissue accumulation and dysfunction that also occurs in parallel with aging (174, 175). Indeed, inflamed adipose tissue attracts macrophages with cell-surface characteristics similar to M2 alternatively activated cells—often assumed to be anti-inflammatory. However, despite their cell-surface phenotype, these cells are potent producers of inflammatory cytokines in adipose tissue, and likely drive age- and obesity-associated inflammation (176–179). Thus, the M1/M2 paradigm for macrophages is likely to be an over-simplification (180, 181). It is unknown if other, primarily tissue-resident cells, are affected by adipose tissue dysfunction, but with aging, the number and function of dendritic cells have been reported to decrease in the skin and mucosal membranes (170). There is also an age-associated increase in myeloid-derived suppressor cells—a heterogeneous population of granulocytes, macrophages, and dendritic cells—that may impair aspects of immune function by producing reactive oxygen species and inhibitory cytokines (182).

Within the adaptive immune system, there are substantial changes to the numbers, function, and phenotype of T cells with aging. Among the broad population of CD4^+^ T-helper cells, aging is associated with a predominance of Th2 (i.e., IL-4 and IL-10), and Th17-producing cells (i.e., IL-17-producing cells that are associated with autoimmune disease), whereas there is a decline in cells with a Th1 profile [i.e., IFN-gamma- and tumor necrosis factor-alpha (TNF-alpha)-producing cells] (183, 184). With aging, the numbers and proportions of antigen-inexperienced CD4^+^ and CD8^+^ T cells decreases (e.g., CD27^+^CD28^+^CD45RA^+^CD57^−^CD62L^+^CCR7^+^KLRG1^−^ naïve cells) (185, 186). In parallel, the numbers and proportions of antigen-experienced CD4^+^ and CD8^+^ T cells increases (e.g., CD27^−^CD28^−^CD45RA^+^CD57^+^CD62L^−^CCR7^−^KLRG1^+^ memory cells), and these cells are potent producers of inflammatory cytokines (185, 186). These changes are driven by lower hematopoietic stem cell numbers, thymic involution resulting in reduced output of antigen-naïve T cells, and infection with latent viruses, in particular *Cytomegalovirus* (185, 187). With aging, T cells that express natural killer cell-associated cell-surface proteins (NKT-like cells) also accumulate, exhibiting similar changes to their phenotype, functional properties, and specificities as with the broader population of CD4^+^ and CD8^+^ T cells (171). There is an age-associated decline in the total number of γδ T cells; however age *per se*, in the absence of chronic infections, is associated with a decline in Vδ2 cells (60–80% of γδ T cells), whereas Vδ1 (15–20% γδ T cells) remain stable (188). Some evidence suggests that γδ T cell proliferative responses are impaired with aging, perhaps due to increased susceptibility of Vδ2 cells to apoptosis (189, 190). Natural regulatory T cells increase with aging whereas inducible regulatory T cells decrease, but it is unclear if their function is affected (191). As with T cells, aging is associated with a decline in the numbers and proportions of naïve B cells, an accumulation of memory B cells with limited specificities, and impaired plasma cell antibody production (192).

Several robust and accepted hallmarks of immunosenescence have been established, especially within the adaptive immune system. For example, low numbers and proportions of naïve T cells (in particular CD8^+^ T cells) and high numbers and proportions of memory T cells (especially late-stage differentiated CD8^+^ T cells) are well established biomarkers (185, 186, 193, 194). In addition, a cluster of parameters, revealed in longitudinal studies of octogenarians and nonagenarians from an isolated population in Sweden, have been referred to as the immune risk profile (195–197). Biomarkers included low numbers and proportions of B cells, high numbers and proportions of late-stage differentiated CD8^+^ T cells (i.e., CD27^−^CD28^−^), poor T cell proliferation in response to mitogens, a CD4:CD8 ratio of <1.0, infection with *Cytomegalovirus* and high plasma IL-6, which together, predicted greater all-cause mortality at 2-, 4-, and 6-year follow-up (195, 197–200). Indeed, the age-associated increase in systemic inflammation, referred to as “inflammageing” is another principle observation among aging and longevity studies (201, 202). Subsequently, high levels of IL-6, TNF-alpha, and C-reactive protein, have been associated with shorter survival (203–205). Overall, it is well established that elderly individuals exhibit impaired immune responses to *in vivo* challenge with novel antigens (143, 206, 207) and these individuals are subsequently thought to be at increased risk of infection. Encouragingly however, as outlined in Part B: “Regular Physical Activity and Frequent Exercise Augment Aspects of Immune Competency Across the Lifespan” exercise can be a potent stimulus of immune function, including the response to vaccination, and some evidence suggests that exercise might delay or limit the age-associated decline in immune competency.

### Relationships Between Exercise and Regular Physical Activity With Hallmarks of an Aging Adaptive Immune System

As summarized in Part B: “Regular Physical Activity and Frequent Exercise Augment Aspects of Immune Competency Across the Lifespan” many studies have examined the influence of regular physical activity or frequent structured exercise on the function of the adaptive immune system with aging (163–165). Here, we focus on recent studies that have examined relationships between exercise, physical activity or cardiorespiratory fitness, and the numbers and proportions of CD4^+^ and CD8^+^ naïve and memory T cells, as hallmarks of immunosenescence. Indeed, a small number of studies have investigated whether the characteristics of the T cell pool are influenced by an active lifestyle. There is a larger body of evidence in young adults, typically between 18 and 30 years, compared to older adults, hereafter considered as being over 40 years of age due to the characteristics of studies published to date. If an active lifestyle can be linked with a smaller accumulation of memory T cells, and a smaller decline in naïve T cells, then in young adults, one interpretation might be that exercise prevents, or at least delays immunosenescence, whereas in older adults, these associations could be interpreted as countering or limiting the development of an age-associated immune profile.

#### Experimental Evidence in Young People: Can Exercise Prevent or Delay Aging of the Adaptive Immune System?

The characteristics of the T cell pool have been examined in 16 young adults (50% male; 18 ± 2 years) classified as being very active (well-trained soccer players self-reporting 9–12 h of exercise per week) and compared to 16 young adults (50% male; 19 ± 2 years) classified as being untrained (individuals self-reporting 2–3 h of exercise per week). Untrained individuals showed the highest proportions of CD4^+^ and CD8^+^ memory T cells, and the lowest proportions of CD8^+^ naïve T cells, defined on the basis of CD57 and CD28 expression (208). Although these results suggest that regular exercise might limit the age-associated accumulation of memory T cells and decline in naïve T cells, the effects were strongly influenced by sex: only untrained males exhibited high proportions of memory T cells and low proportions of naïve cells compared to trained males, and these effects were driven by changes in the CD4^+^ T cell pool (208). Extending these findings by examining a female-only population of young adults, the same authors compared 13 well-trained soccer players (self-reporting around 12 h of exercise per week; 20 ± 2 years) to 13 untrained controls (self-reporting around 3 h of exercise per week; 21 ± 2 years) (209). Trained females exhibited a greater proportion of CD8^+^ naïve T cells compared to untrained females, but these associations did not remain statistically significant after controlling for body fat percentage (trained 21.7 ± 4.0 versus untrained 25.1 ± 4.1%, *p* < 0.05) (209). Indeed, very few studies have considered whether relationships between an active lifestyle and markers of an aging immune system could be influenced by other factors, such as body composition. Recently, a very small study of 15 males aged 30 ± 4 years, categorized participants using a combination of gold standard methods for measuring physiological and lifestyle variables (210). Three groups were formed (sedentary, active, and very active) on the basis of objectively assessed habitual physical activity, directly measured cardiorespiratory fitness, and body composition assessed with dual energy X-ray absorptiometry (210). This work showed that sedentary individuals had higher proportions of memory CD4^+^ T cells expressing CD45RO and PD-1, supporting the results of other published studies that have not taken body composition into consideration.

It should be emphasized, that among younger adults in particular, mixed results have been reported when investigating links between an active lifestyle and hallmarks of an aging immune system. Most investigations have been cross-sectional in design, or have made observations between groups over short periods. For example, one study has shown that national standard triathletes (age range 18–36 years, *n* = 19), appear to exhibit impaired thymic output, assessed by T cell receptor excision circle levels (211). Among CD4^+^ and CD8^+^ T cells, these athletes had lower absolute numbers of naïve cells and higher absolute numbers of memory cells compared to age-matched less active controls (*n* = 16), as shown by CD45RA, CD45RO, and CD27 expression (211). Similar observations have been made by examining individuals in the third decade of life, showing that endurance athletes, compared to less active controls, appear to exhibit slightly larger accumulations of memory T cells and slightly fewer naïve T cells, defined by CD45RA and CCR7 expression (212). Thus, it appears that among younger individuals (i.e., less than 40 years of age), if exercise is undertaken at very extreme volumes, such as more than 5–10 times the amount of physical activity recommended each week (i.e., 12–25 h per week), this might contribute toward a small decline in naïve T cells and a small increase in memory T cells. It is possible that these changes are due to reactivation of latent viruses, which could be independent of immune function, and driven by exercise-induced adrenergic activity, oxidative stress and inflammatory cytokines (48–50). However, these results might also be explained by fluctuations in cell numbers and cell sub-populations in peripheral blood over time. Such changes have been interpreted as being linked to exercise training load (213, 214), but it is also conceivable that these changes occur due to seasonal variation, as has been shown in non-exercise contexts (215, 216).

#### Experimental Evidence in Older People: Can Exercise Limit or Counter Aging of the Adaptive Immune System?

Although most studies have examined associations between biomarkers of an aging adaptive immune system in young adults, other studies have made measurements across a broader range of ages. For example, one study examined 102 men ranging in age from 18 to 61 years (mean 39 ± 6 years) (217). It was shown that the proportion of the CD4^+^ and CD8^+^ T cell pool comprising of memory cells (defined as KLRG1^+^CD57^+^ or KLRG1^+^CD28−) was inversely correlated with cardiorespiratory fitness, which is largely indicative of an active lifestyle (217). The age-associated decrease of naïve T cells (defined as KLRG1^−^CD57^−^ or KLRG1^−^CD28^+^) and increase in memory T cells did not withstand statistical adjustment for cardiorespiratory fitness, but remained significant after adjusting statistically for body composition and *Cytomegalovirus* infection (217). Thus, it was concluded that fitter individuals exhibit a smaller age-associated decline of naïve T cells and a smaller accumulation of memory T cells.

As with work examining relationships between an active lifestyle and hallmarks of an aging adaptive immune system in young and middle-aged adults, similar associations have been shown in an older population of 61 men aged 65–85 years (218). In this study, participants self-reported to be “untrained” (*n* = 15), or to lead a “moderate” training lifestyle (*n* = 16; taking part in team sports or running less than 6 km two to three times per week), or an “intense” training lifestyle (*n* = 15; running approximately 10 km at least 5 days per week). These categories were confirmed with a validated physical activity questionnaire and by measurement of cardiorespiratory fitness. Both training groups exhibited a lower proportion of CD4^+^ and CD8^+^ memory cells (defined as CD45RA^+^CCR7^−^), but these associations were largest and only statistically significant among men leading an “intense” training lifestyle, suggesting a dose–response effect of exercise. Although findings were less clear when examining other cell subpopulations based on CD45RA and CCR7 expression, and there were no effects of exercise when examining CD28^−^ cells, men in the “trained” groups had T cells with the longest telomeres (218). Another recent study compared 125 adults (55–79 years of age) who maintained a high level of cycling throughout life to 75 age-matched inactive controls (219). Within the CD4+ and CD8+ T cell pool, the frequency of naive cells (defined as CD45RA+) was greater, and the frequency of memory cells (defined as CD45RA-) was lower among cyclists. Extended phenotyping revealed that CD4+ and CD8+ CCR7-CD45RA+ accumulation was less among cyclists, but no differences were found for CD28-CD57+ cells. Cyclists also exhibited higher frequencies of recent thymic emigrants and regulatory B cells, lower Th17 polarization, and in plasma, higher IL-7 and lower IL-6 (219). Despite these findings, suggesting a beneficial effect of leading an active lifestyle on immunosenescence among older adults, there is some inconsistency in the literature. For example, comparing elderly athletes (*n* = 12, approximately 74 years of age) to less active age-matched controls (*n* = 26), there were no differences in thymic output, the proportions of naïve or memory CD4^+^ and CD8^+^ T cells (defined with CD45RA and CCR7 expression), or T cell activation in response to anti-CD3 stimulation (212). Most investigations of T cell immunosenescence and lifestyle among healthy elderly adults have had cross-sectional study designs. Longitudinal studies, or randomized and controlled trials of exercise training are lacking and might yield promising results. For example, one study has compared 6 months of exercise training in men and women (*n* = 28, aged 61–76 years) to a similar group who maintained their current lifestyle (*n* = 20, aged 62–79 years) (220). Using a simple immunophenotyping strategy, the results showed that the proportion of CD4^+^ T cells expressing CD28 increased in the exercise group after 6 months, but not in those who maintained their lifestyle (220).

#### Summary of Experimental Evidence

In summary, evidence shows that the characteristics of the T cell pool appear to be influenced by leading an active lifestyle, determined by exercise training, physical activity level, or cardiorespiratory fitness. It seems that among both the young and elderly, an active lifestyle is generally linked to lower numbers and proportions of memory T cells and higher numbers and proportions of naïve T cells (10). This summary is partly supported by a recent systematic review, concluding that regular structured exercise increases the number of naïve T cells in peripheral blood at rest (221). Altogether, findings from recent studies examining relationships between an active lifestyle and the characteristics of the T cell pool—as robust and accepted biomarkers of immunosenescence—support observations from some cross-sectional and longitudinal studies, showing that other measures of immune competency, which typically decline with aging, can be improved with physical activity or regular structured exercise (163–165). However, further research is needed in this area that employs precise lifestyle measurements (e.g., using wearable technology to assess physical activity, and dual energy X-ray absorptiometry to measure body composition) and more robust measurements of immune competency (e.g., absolute cell counts rather than proportions, measurements of cell function, and *in vivo* antigen challenges) while controlling for factors that drive immunosenescence (e.g., inflammation and *Cytomegalovirus* infection).

#### Mechanisms

Links between a physically active lifestyle with lower numbers or proportions of memory T cells, and higher numbers or proportions of naïve T cells, have been hypothesized as being driven by the acute effects of exercise bouts. For example, it has been suggested that repeated bouts of exercise might prevent or delay immunological aging by limiting the accumulation of CD4^+^ and CD8^+^ antigen-experienced memory T cell clones, repopulating blood with antigen-inexperienced naïve T cells (11, 12). In this hypothesis, it is proposed that memory T cells are frequently mobilized into blood during regular bouts of exercise, followed by an extravasation to peripheral tissues, where these cells are exposed to pro-apoptotic stimuli, such as reactive oxygen species, glucocorticoids, and cytokines (11, 12). Subsequently, it is proposed that the number of naïve T cells increases as part of a negative feedback loop governing the number of naïve and memory cells in the immune system, which is bolstered by exercise-induced thymopoiesis and extrathymic T cell development (11, 12). Supporting the mechanisms proposed in this hypothesis, many investigations have shown that memory T cells are mobilized into the circulation during exercise, followed by extravasation out of the bloodstream in the hours following (81, 123). In addition, studies in mice show that lymphocyte apoptosis occurs post-exercise in tissues thought to be the homing destination of mobilized cells (222). Although some T cells mobilized by exercise might be resistant to apoptosis, given that *Cytomegalovirus*-specific CD8^+^ T cells express high levels of Bcl-2 (223), other work has shown that *Cytomegalovirus*-specific CD8^+^ T cells, are equally as susceptible to Fas-induced apoptosis as the total pool of CD8^+^ T cells (224). Further, irrespective of virus specificity, studies have shown that T cells expressing cell-surface proteins such as CD57 and KLRG1 are more susceptible to H_2_O_2_-induced apoptosis than total lymphocytes and naïve T cells (225, 226). Thus, the concept of exercise directly countering memory T cell accumulation is supported by evidence from human and animal studies.

It is unknown whether triggering apoptosis among expanded clones of memory T cells specific for viruses such as *Cytomegalovirus* is advantageous. For example, in a transplant setting, *Cytomegalovirus* disease occurs when T cells fail to provide antiviral control (227) and a robust pro-inflammatory response to *Cytomegalovirus* has been associated with longer survival in the elderly (228). However, it remains to be determined what proportion of the T cell pool needs to be specific for *Cytomegalovirus* to limit viral reactivation. Infection with *Cytomegalovirus* results in approximately 10% of the CD4^+^ and CD8^+^ T cell pool becoming specific for this virus (229), although large inter-individual differences exist. For example, it has been reported that 23% of the CD8^+^ T cell pool can become specific for a single *Cytomegalovirus* epitope (223). Traditionally, it has been considered disadvantageous for such a large proportion of the T cell pool to be specific for one virus. This view is linked to another age- or infection-associated change that occurs in parallel—a fall in the numbers and proportions of naïve cells—which has been interpreted as limiting capacity to engage novel antigens. These interpretations are based upon two assumptions. First, there is an upper limit to the size of the immune system, and second, thymic output is negligible after adolescence (230). Thus, it has been proposed that antigen-inexperienced naïve T cells could be “used up” due to ongoing differentiation into antigen-experienced memory T cells that “fill up” immunological “space” (230). It has also been proposed that this accumulation of antigen-experienced memory T cells leads to “squeezing out” of T cells targeting less dominant viruses leading to loss of viral control (231). This concept of a fixed amount of immunological space has since been debated (232, 233) and thymic output is now known to persist, albeit reduced, up until around 70 years of age (234). However, even if removal of some memory T cells is not essential for maintaining an effective T cell pool, assuming these cells contribute to systemic inflammation, their removal might limit “inflammageing” (230). Despite uncertainties over the susceptibility of memory T cells to undergo apoptosis, or whether it is advantageous to stimulate their removal, it seems that exercise-induced immune cell death in the tissues has relevance to other processes. For example, apoptotic cells and cell debris stimulates hematopoietic stem cell mobilization into blood (95) perhaps promoting trafficking to the thymus or extrathymic sites facilitating output of naïve T cells (235). Additional support for exercise stimulating production of naïve T cells is provided by work showing that contracting skeletal muscle produces IL-7 (236) which might increase thymic mass and function (237).

A physically active lifestyle might also counter T cell immunosenescence indirectly, perhaps by limiting adipose tissue accumulation and dysfunction that occurs with aging and obesity (174, 238, 239). Indeed, obesity has been linked with impaired lymphocyte proliferation (240), shorter leukocyte telomere length (241), and a skewing of the T cell pool toward a regulatory and Th2-phenotype (242). In addition, large expansions of differentiated αβ T cells and γδ T cells have been shown among people with obesity, with γδ T cells exhibiting impaired antiviral function (243–245). It is generally accepted that repeated stimulation with antigens from *Cytomegalovirus* drives immunosenescence (185, 186, 193, 194). With obesity, adipose tissue is the primary source of pro-inflammatory cytokines and reactive oxygen species (174, 246) which can reactivate *Cytomegalovirus* directly (48, 49). Thus, exercise might limit T cell immunosenescence by decreasing visceral and subcutaneous adipose tissue (238), providing a potent anti-inflammatory and anti-oxidative stimulus (247, 248). In turn, lower systemic inflammation and better redox balance might limit viral reactivation, reducing stimulation with antigens from viruses such as *Cytomegalovirus*. In addition, T cell dysfunction might also be prevented, in part, by limiting reactive oxygen species production (249).

In summary, leading a physically active lifestyle appears to limit the age-associated changes to the cellular composition of the adaptive immune system, but the mechanisms are yet to be determined. Exercise might counter the expansion of memory T cells directly, which is desirable assuming these cells contribute to systemic inflammation and not all are required to control latent viruses. Limiting the expansion of memory T cells also assumes the “size” of the immune system is fixed, the capacity to produce antigen-naïve T cells is limited, and these constraints contribute to immune decline in the elderly. However, exercise might affect memory T cell accumulation indirectly, by reducing viral reactivation, or preventing T cell senescence, by controlling adipose tissue deposition and dysfunction that drives inflammation and oxidative stress with aging and obesity.

## Concluding Remarks

Contemporary evidence from epidemiological studies shows that leading a physically active lifestyle reduces the incidence of communicable (e.g., bacterial and viral infections) and non-communicable diseases (e.g., cancer), implying that immune competency is enhanced by regular exercise bouts. However, to this day, research practice, academic teaching, and even physical activity promotion and prescription continues to consider a prevailing myth that exercise can temporarily suppress immune function. We have critically reviewed related evidence, and conclude that regular physical activity and frequent exercise are beneficial, or at the very least, are not detrimental to immunological health. We summarize that (i) limited reliable evidence exists to support the claim that exercise suppresses cellular or soluble immune competency, (ii) exercise *per se* does not heighten the risk of opportunistic infections, and (iii) exercise can enhance *in vivo* immune responses to bacterial, viral, and other antigens. In addition, we present evidence showing that regular physical activity and frequent exercise might limit or delay immunological aging. We conclude that leading an active lifestyle is likely to be beneficial, rather than detrimental, to immune function, which may have implications for health and disease in older age.

## Author Contributions

JC and JT contributed equally toward literature searching and retrieval, the ideas and interpretation of the studies described, drafting and revision of the manuscript, and approval of the final version to be published. JT and JC both agreed to be accountable for the work in ensuring that questions related to the accuracy or integrity of any part of the work are appropriately investigated and resolved.

## Conflict of Interest Statement

The authors declare that the research was conducted in the absence of any commercial or financial relationships that could be construed as a potential conflict of interest.

## Footnotes

^1^“Physical activity” refers to activities undertaken during leisure time, at home, as part of employment, or for transport purposes. “Exercise” is a component of physical activity within the leisure time domain and refers to physical activities that are planned, structured, repetitive, and undertaken for the purpose of improving or maintaining components of physical fitness and/or sporting performance. When individuals are referred to as being “active” or “inactive,” the description infers that these people undertake (or fail to undertake) a defined level of exercise or physical activity (e.g., age-specific physical activity recommendations, such as those published by the World Health Organisation). In this review, the term “exercise” will generally be used to describe the effects that active behaviours have on immune competency. Individuals described as being “sedentary” accumulate prolonged periods of behaviour eliciting low energy expenditure (e.g. sitting and lying).
